# Multigene expression of protein complexes by iterative modification of genomic Bacmid DNA

**DOI:** 10.1186/1471-2199-10-87

**Published:** 2009-09-02

**Authors:** Rob J Noad, Meredith Stewart, Mark Boyce, Cristina C Celma, Keith R Willison, Polly Roy

**Affiliations:** 1 Department of Infectious and Tropical Diseases, London School of Hygiene and Tropical Medicine, Keppel Street, London, WC1E 7HT, UK; 2 Section of Cell and Molecular Biology, Institute of Cancer Research, 237 Fulham Road, London SW3 6JB, UK; 3 Current address: Department of Pathology and Infectious Diseases, Royal Veterinary College, Hawkshead Lane, Hatfield AL9 7TA, UK

## Abstract

**Background:**

Many cellular multi-protein complexes are naturally present in cells at low abundance. Baculovirus expression offers one approach to produce milligram quantities of correctly folded and processed eukaryotic protein complexes. However, current strategies suffer from the need to produce large transfer vectors, and the use of repeated promoter sequences in baculovirus, which itself produces proteins that promote homologous recombination. One possible solution to these problems is to construct baculovirus genomes that express each protein in a complex from a separate locus within the viral DNA. However current methods for selecting such recombinant genomes are too inefficient to routinely modify the virus in this way.

**Results:**

This paper reports a method which combines the lambda red and bacteriophage P1 Cre-recombinase systems to efficiently generate baculoviruses in which protein complexes are expressed from multiple, single-locus insertions of foreign genes. This method is based on an 88 fold improvement in the selection of recombinant viruses generated by red recombination techniques through use of a bipartite selection cassette. Using this system, seven new genetic loci were identified in the AcMNPV genome suitable for the high level expression of recombinant proteins. These loci were used to allow the recovery two recombinant virus-like particles with potential biotechnological applications (influenza A virus HA/M1 particles and bluetongue virus VP2/VP3/VP5/VP7 particles) and the mammalian chaperone and cancer drug target CCT (16 subunits formed from 8 proteins).

**Conclusion:**

**1**. Use of bipartite selections can significantly improve selection of modified bacterial artificial chromosomes carrying baculovirus DNA. Furthermore this approach is sufficiently robust to allow routine modification of the virus genome. **2**. In addition to the commonly used *p10 *and polyhedrin loci, the *ctx, egt, 39k, orf51, gp37, iap2 *and *odv-e56 *loci in AcMNPV are all suitable for the high level expression of heterologous genes. **3**. Two protein, four protein and eight protein complexes including virus-like particles and cellular chaperone complexes can be produced using the new approach.

## Background

The baculovirus *Autographa califonica multiple nucleopolyhedrosis virus *(AcMNPV) is routinely used to express proteins in eukaryotic cells for structural, biochemical and vaccine studies [1]. The AcMNPV genome is circular dsDNA (~134 kb) and contains regions of highly repetitive DNA elements and genes in both strands [2]. This genomic DNA can be propagated in *Escherichia coli *as a bacmid, and genes can be inserted by *Tn7 *transposase based transposition into the virus DNA [3]. Transfection of the modified bacmid DNA into virus susceptible insect cells results in recovery infectious virus expressing the recombinant protein corresponding to the inserted gene [3]. Alternate approaches in which homologous recombination is carried out in insect cells can also be used to generate recombinant baculoviruses [4,5].

One of the advantages of the baculovirus system is its utility for the co-expression of multiple genes that encode protein complexes [6-12]. This is important, as many critical functions of living cells are carried out by multi-subunit protein complexes which are naturally present at low abundance. One approach for the baculovirus mediated co-expression of multiple genes is the insertion of expression cassettes as tandem or inverted repeats at the polyhedrin locus in the viral genomic DNA [6-9,12,13]. This approach ensures that every cell in the culture expresses the genes encoding recombinant proteins in the same ratio and results in consistent yields of recombinant protein complex [14]. However, this approach has two major limitations. Firstly, there is a practical limit to the number of genes that can be inserted into one transfer vector in terms of vector size. In practice, this means that it is rarely possible to express more than four proteins from a single locus. In addition, baculovirus expresses proteins that promote homologous recombination [15-17], therefore viruses that contain large amounts of repeated sequences, such as common promoters and terminators, are prone to rearrangement and recombination [11,18,19]. A potential solution to these problems would be the production of viral genomes in which foreign genes are each expressed from a different genetic locus, removing the need for large transfer vectors with highly repetitive sequences. However, to date, most baculovirus expression experiments have been carried out with one of two loci (polyhedrin and p10). There have also been reports of *v-cath *[10,11] being used to express heterologous proteins, but the expression of foreign genes from other loci in the genome is largely uncharacterised. Therefore, before any multi-locus approach to express genes for larger complexes could be pursued it would be necessary to characterise foreign gene expression at alternative loci within the AcMNPV genome.

Recently baculovirus research has benefited from use of the lambda red recombination [20] approach for selective knockout of viral genes [21-28]. However, the potential for this technique to engineer high level expression of foreign genes from different genetic loci within the baculovirus genome has not been examined. In this report, we describe high level expression of foreign genes from seven loci within the AcMNPV genome not previously characterised for this purpose. Furthermore, we demonstrate a functional multi-locus system for the expression of multiple foreign genes from a single baculovirus genome. In this system single protein expression cassettes are inserted efficiently at different loci within the viral genome using lambda red recombination. As single-gene insertions are thus distributed throughout the AcMNPV genome, the problems of transfer vector size and highly repetitive inserts are overcome. We demonstrate the utility of this system for the expression of complexes with up to eight subunits using influenza A VLPs (2 proteins), bluetongue VLPs (4 proteins) and the essential mammalian chaperonin complex CCT (8 proteins) as examples.

## Results

### A bipartite selection system allows significant increase in the efficiency of recombinant bacmid recovery

**Figure 1:**
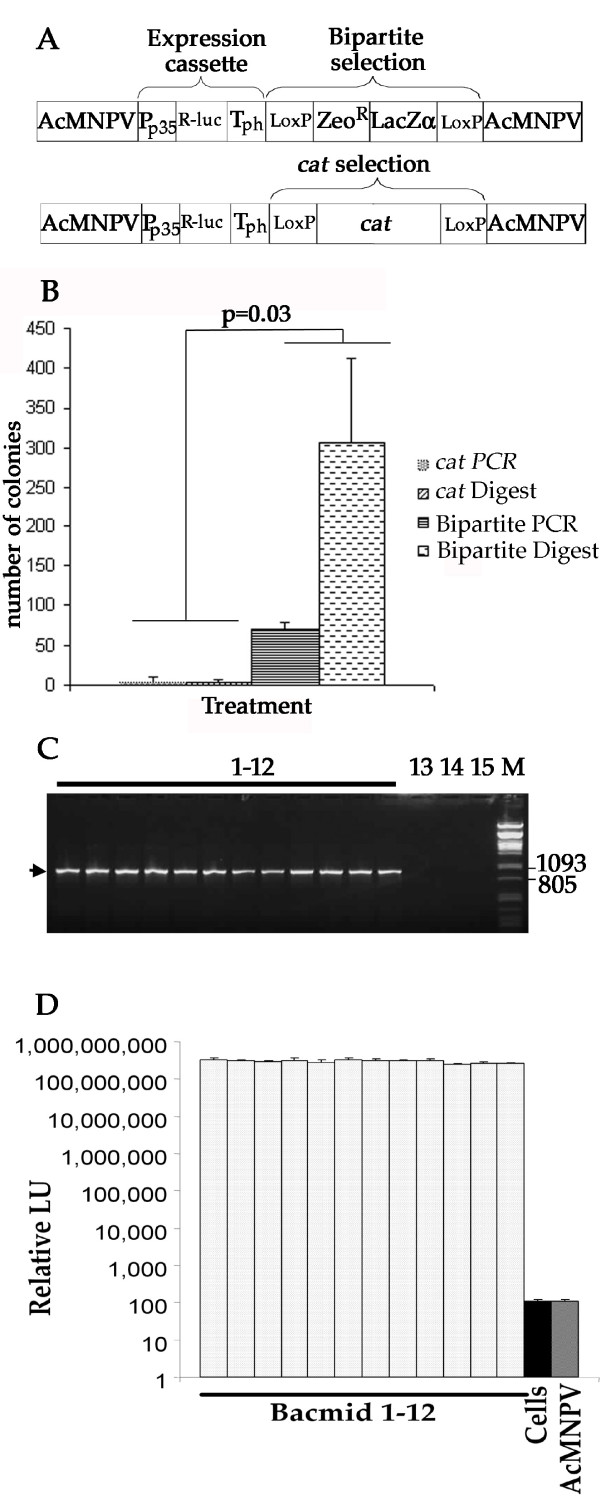
**Bipartite selection provides significant increase in selection efficiency for lambda red Bacmid recombination**. A) Two recombination cassettes were designed, both with baculovirus flanking sequences (AcMNPV), p35 promoter (P_p35_), Renilla Luciferase (R-luc) and polyhedrin polyadenylation sequences (T_ph_) and LoxP sites flanking a bacterial selectable marker. For bipartite selection (top) a Zeocin resistance gene (ZeoR) and LacZα marker were incorporated. For single marker selection (bottom) the *cat *gene, conferring chloramphenicol resistance, was incorporated. B) The number of positive colonies following red recombination using 30 ng of each recombination cassette (~12.58 fmol), generated either by PCR amplification or restriction enzyme digestion, and BACmid DNA. Each transformation was carried out in triplicate, error bars indicate the standard deviation of the results. The data for the two different selections were clustered and a t-test used to confirm that the results were statistically significant (p = 0.03). C) PCR confirming recombination and correct target site 12 independent bacmid recombinants (lanes 1-12). One primer annealed to genomic DNA flanking the target insertion site and the other to sequence inside the recombination cassette. Only recombination at the correct genetic locus would produce the PCR product (arrowed). Lane 13, no template PCR reaction; Lane 14, bMON14272 Bacmid template DNA; Lane 15 plasmid containing the entire DNA used for recombination as template. D) Renilla luciferase activity at 48 hours post infection in cell lysates from cells infected with passage 2 of recombinant bacmids 1-12 from B. Cells and AcMNPV indicate background activity in lysates from uninfected and unmodified bacmid infected cells, respectively.

Lambda red recombination [20] has been widely used to introduce knockout mutations in AcMNPV [21-28]. In the majority of these experiments a chloramphenicol resistance gene has been used as a selectable marker. In our hands, although this system worked, the speed with which experiments could be completed was hampered by the number of false positive colonies arising due to the low levels of antibiotic used in selection of the low copy bacmid. Also, it was not possible to introduce multiple modifications into the virus genome without either accumulating antibiotic resistance genes or performing another round of lambda red recombination and selecting for virus which had lost the selectable marker. To overcome these problems we designed a new bipartite selection cassette based on *LacZα *fragment and Zeocin resistance gene flanked by modified loxP recombination sites (Fig. 1A). Using this system, sufficient Zeocin was used to reduce, but not eliminate, background colony growth and recombinants were selected based on blue colony phenotype in the presence of IPTG and X-gal.

To assess the relative efficiency of recovering recombinants using the chloramphenicol and bipartite selections, recombination competent *E. coli *containing unmodified AcMNPV bacmid were electroporated with 30 ng (~12.5 fmol) linear DNA containing each selection cassette (Fig. 1B). The efficiency of recombination using PCR derived and plasmid derived, restriction enzyme released, linear DNA was compared. PCR primers were designed such that the ends of the PCR products corresponded to the ends of the restriction enzyme released fragments. The bipartite selection resulted in a 21 fold increase in the number of positive colonies for PCR derived DNA and an 88 fold increase for restriction enzyme released plasmid DNA compared to the single antibiotic selection. These differences were significant (t-test, p = 0.03). To avoid repeated PCR of coding sequences, and to take advantage of the increased efficiency of recombination, linear DNA derived by restriction digestion of plasmid DNA was used for subsequent experiments.

PCR was carried out on bacmid DNA purified from positive colonies from the bipartite selection to confirm correct insertion of the expression construct. Primers complementary to the zeocin resistance gene and the AcMNPV DNA flanking the correct insertion site were used. PCR product was only produced where correctly targeted recombination had occurred. DNA for twelve separate bacmids were tested using this method (Fig. 1C, lanes 1-12), all were positive for the PCR product, indicating correctly targeted recombination. In contrast, neither bacmid DNA alone, nor plasmid DNA containing the DNA fragment used for recombination was able to act as template to produce the PCR product (Fig. 1C, lanes 14 and 15 respectively). To test the viability of recombinant baculovirus, the same twelve PCR positive bacmid clones were transfected into *Sf*9 cells and passaged twice, then Renilla luciferase activity in cells infected with each of the recombinants was assayed at 48 hours post infection. Cells infected with each of the twelve recombinant viruses had Renilla luciferase activities that were 10^6 ^fold above background (Fig. 1D). Based on these data the bipartite selection was used for further studies. The genetic locus used for these initial studies was the *p10 *locus, which was modified to remove native *p10 *promoter sequences.

### Cre mediated removal of the selectable marker allows multiple rounds of recombination with the same bipartite selection

**Figure 2:**
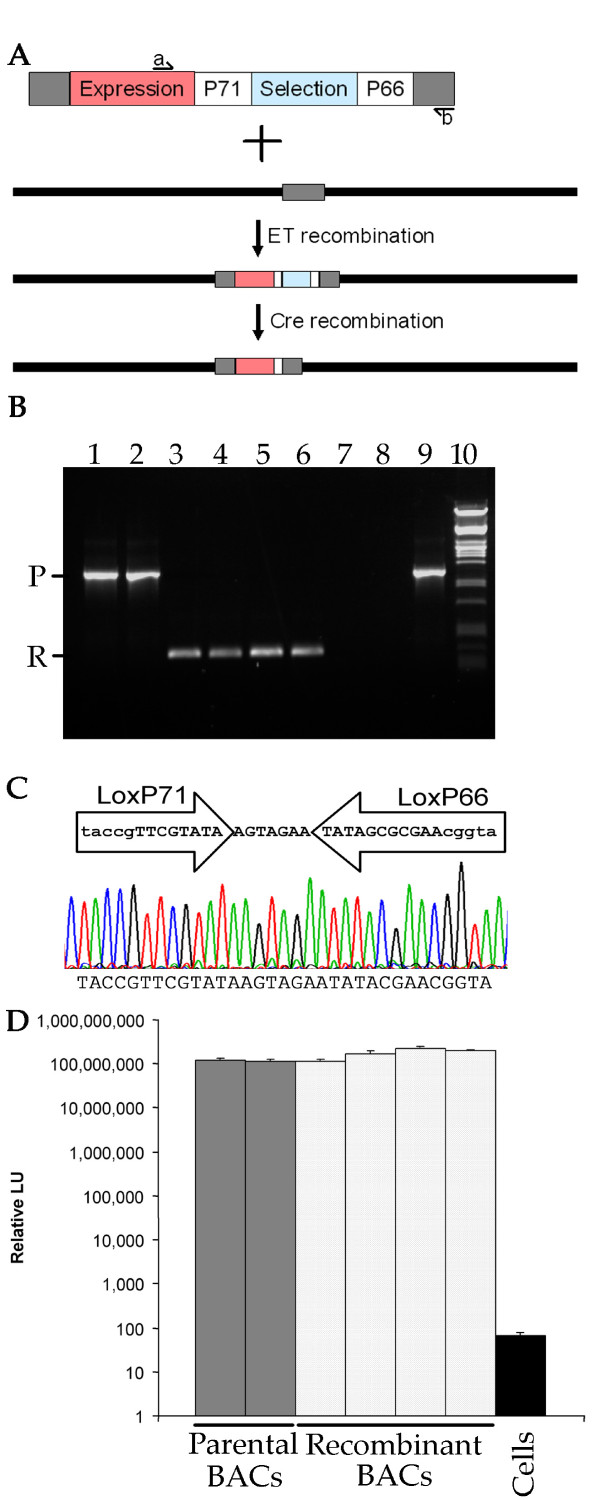
**Selective removal of marker genes**. A) Cartoon showing strategy for Recombineering of an expression cassette (expression) into the AcMNPV Bacmid DNA and selective removal of only the marker genes (selection) by Cre mediated recombination. B) PCR using primers labelled a and b in A using: Lanes 1-2, two independent bacmid recombinants following red recombination; Lanes 3-6, four independent recombinants following Cre mediated recombination to remove the bacterial selectable markers; Lane 7, no template; Lane 8, unmodified bacmid DNA template; Lane 9, plasmid DNA template containing the selectable marker cassette; Lane 10, DNA marker. PCR products corresponding to the sizes predicted for the parental and recombinant products of the Cre mediated recombination are labelled P and R respectively. C) Sequencing trace file of a representative recombinant from the PCR analysis in B confirming the presence of a defective *lox*P incorporating the *loxP71 *and *loxP66 *arms that render the recombinant incapable of undergoing further Cre-mediated recombination. D) Renilla luciferase activity of the parental and recombinant bacmids from B when transfected into insect cells. Renilla luciferase activity was assayed at 48 hours post infection after on passage 2 of the recombinant virus. Background activity from uninfected cells is labelled Cells.

In order to engineer multiple insertions in the AcMNPV genome, the bipartite selection cassette was flanked by modified loxP sites. These sites incorporated both the *lox66 *and *lox71 *mutations that limit Cre mediated recombination to a single round [29], and a mutation in the spacer reducing homology to the wildtype *loxP *site. Cre mediated recombination would thus result in the removal of the bipartite selectable marker and inactivation of the *lox *recombination sites but leave behind the baculovirus expression cassette (Fig. 2A). To confirm this strategy could be used successfully to engineer multiple insertions in the bacmid DNA, Cre recombination was used to remove the bipartite marker from bacmid in which the renilla luciferase reporter had been inserted. Bacmid DNA was purified from four putative recombinants and recombination confirmed by PCR using primers flanking the selectable marker (Fig. 2B). All four PCR products were consistent with the size expected for successful Cre recombination. This was further confirmed by sequencing across the modified *loxP *site of the recombinants (Fig. 2C). To confirm that Cre mediated bacmid recombinants remained viable in insect cells, bacmid DNA was transfected into insect cells and luciferase activity assayed after two passages as before. All recombinants had renilla luciferase activity that was equivalent to the parental baculovirus before Cre recombination (Fig. 2D).

### Identification of genetic loci in the baculovirus genome suitable for high level expression of heterologous genes

**Figure 3:**
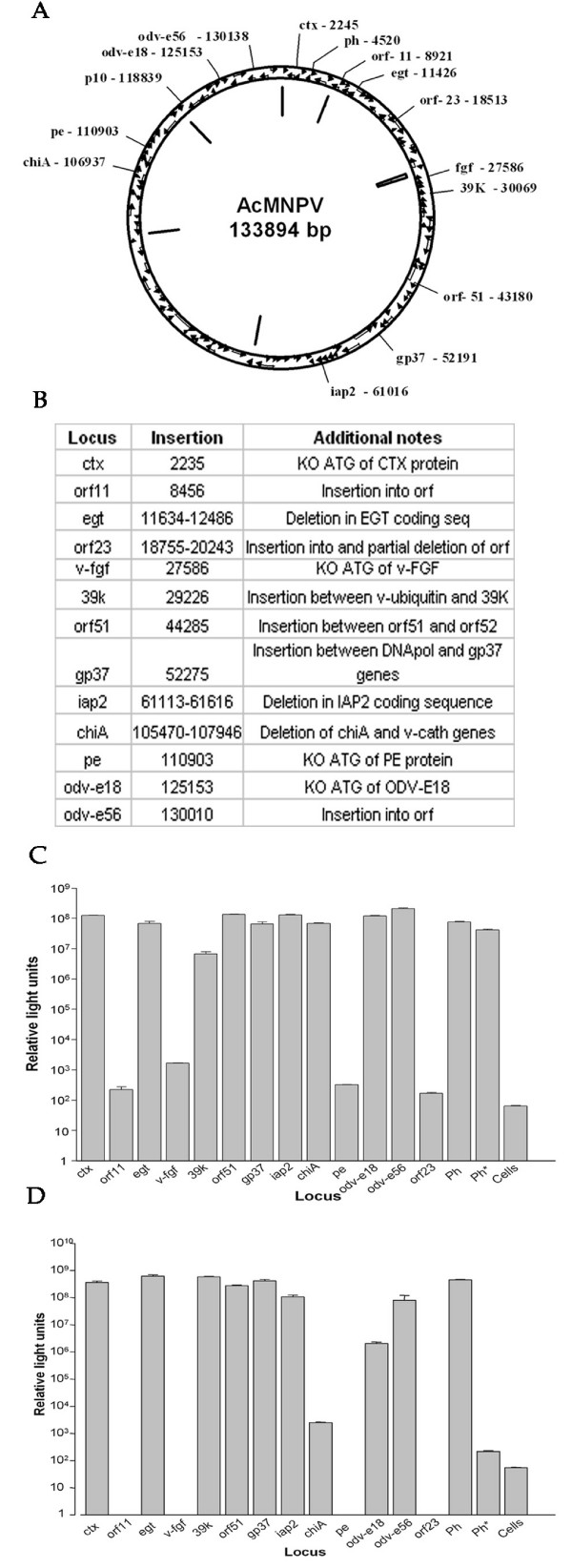
**Identification of additional sites for expression of recombinant proteins in the AcMNPV genome**. A) Genome of AcMNPV showing relative locations of loci used for protein expression. Bars inside the genome indicate position of *hr *sequences. B) Table showing loci used for insertion and any additional changes made to locus. KO indicates knockout. C) Relative renilla luciferase activity at 48 hours post infection with virus modified to contain an additional firefly luciferase expression cassette at each locus indicated. All viruses had the same renilla luciferase expression cassette at the p10 locus under control of the p35 promoter. Error bars indicted the standard deviation of five replicates for each locus. D) Normalised firefly luciferase activity showing relative expression of a polyhedrin promoter-polyhedrin terminator inserted at each locus as indicated. Error bars indicate the standard deviation from 5 replicates for each locus. Firefly luciferase was normalised using the renilla luciferase control expressed from the same genome. Firefly luciferase insertions at the *orf11, v-fgf, pe *and *orf23 *loci were excluded due to renilla luciferase levels more than 2 logs lower than positive control virus. Virus Ph has firefly luciferase at the polyhedrin locus and renilla luciferase at the p10 locus. Virus Ph* has the same p10 renilla luciferase insertion but no firefly luciferase insertion.

To test whether the new selection could be used efficiently at different baculovirus genetic loci a second round of recombination was carried out using one of the bacmids already containing the renilla luciferase gene. A second reporter, firefly luciferase, under control of the polyhedrin promoter was inserted at a total of thirteen different genetic loci (*ctx, orf11, egt, orf23, v-fgf, 39k, orf51, gp37, iap2, chiA, pe, odv-e18 *and *odv-e56*), in each case generating a dual expression baculovirus for Renilla and firefly luciferase (Fig. 3A). Additional changes were made at certain loci to specifically inactivate the expression of the endogenous viral gene (Fig. 3B). Recombinant viruses were passaged twice in *Sf*9 insect cells and on the third passage cells were harvested at 48 hours post infection, lysed and assayed for both firefly and Renilla luciferase activities. Renilla luciferase activity was used as a reference for virus replication and protein expression as all the recombinants had the same P35 promoter driven Renilla luciferase cassette in the *p10 *locus. Expression of firefly luciferase at each locus was compared to a virus carrying firefly luciferase gene at the polyhedrin locus. Of the thirteen loci tested, nine had renilla luciferase activities which were at least 10^5 ^fold above background, and of these eight (*ctx, egt, orf51, gp37, iap2, chiA, odv-e18 *and *odv-e56*) had renilla luciferase activity which was equivalent to that of the parental renilla luciferase only control (Fig. 3C). Thus there was no evidence of a general impairment in virus replication or gene expression for these viruses. In contrast, four insertions (*orf11*, *v-fgf*, *pe *and *orf23*) resulted in virus infections where renilla luciferase activity was within 10 fold of the background and were exluded from further analysis.

Renilla luciferase activity was used as a reference to normalise firefly luciferase activity and obtain a measure of relative expression of the firefly luciferase from each locus (Fig. 3D). Seven loci (*ctx, egt, 39k, orf51, gp37, iap2 *and *odv-e56*) had firefly luciferase activity which was at least 10^6 ^fold higher than background and similar to polyhedrin locus expression of the gene (Fig. 3D). Two viruses (*chiA *and *odv-e18*) had high levels of renilla luciferase but relatively poor firefly luciferase activity suggesting that although these viruses were capable of replication, expression of the polyhedrin promoter driven reporter at this locus was impaired.

There was no clear correlation between the position of a locus within the virus genome and its ability to allow successful high level expression of the foreign reporter gene (Additional file 1: Figure S1). Nor was there any correlation between the level of expression observed for the reporter and that documented for the native gene at the same locus [30].

### Simultaneous co-expression of two and four genes to recover virus-like particles

**Figure 4:**
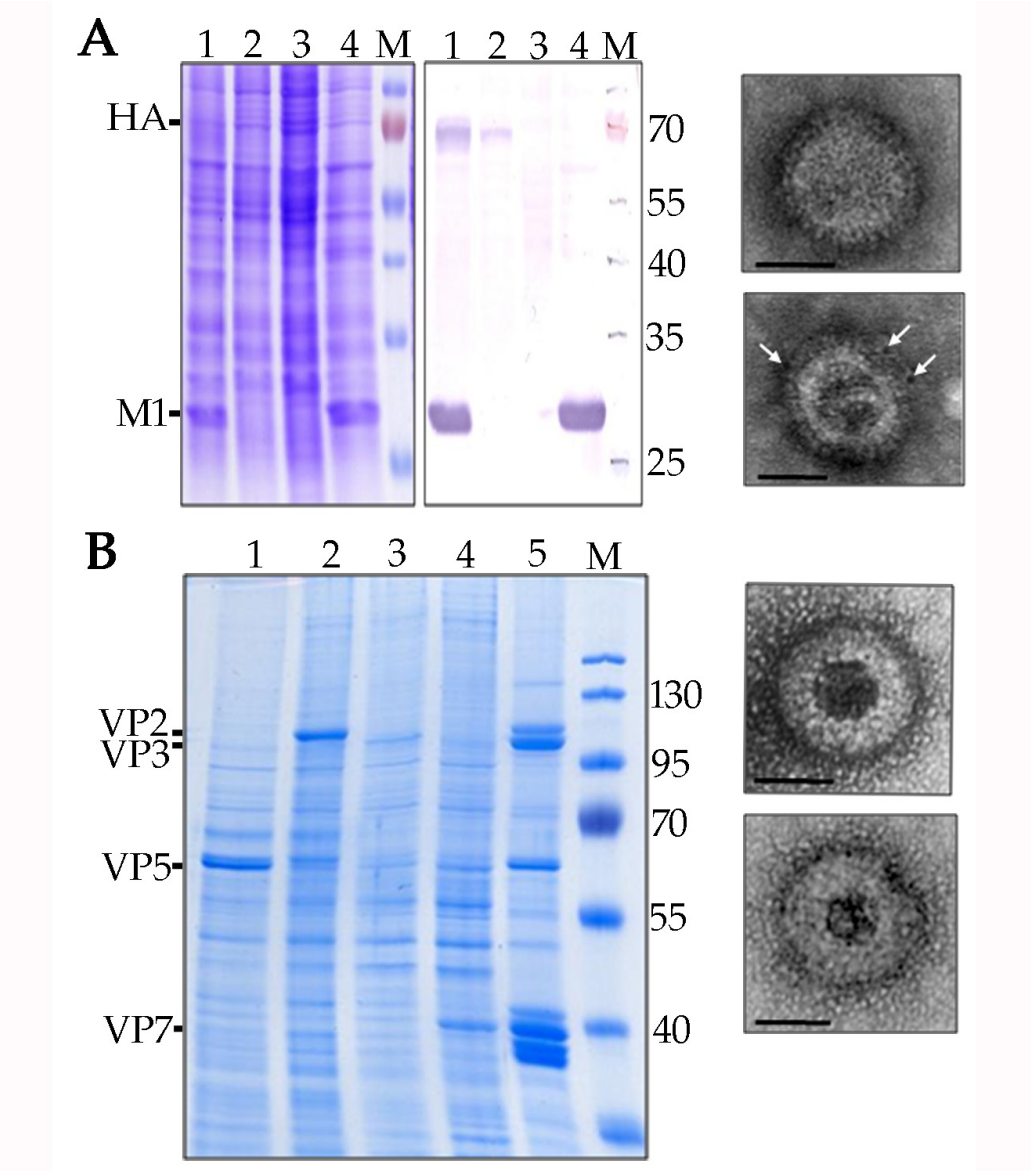
**Expression of virus like particles**. A) Influenza A VLPs. Left and middle, stained SDS-PAGE and immunoblot, respectively, of cells expressing; Lane 1, Influenza HA (*p10*locus) and M1 (*egt *locus); Lane 2, Influenza HA (*p10 *locus) only; Lane 3, uninfected cells; Lane 4, Influenza M1 (*egt *locus). The immunoblot was probed with a polyclonal anti-influenza (H7N7) antibody. Right panel negative stain electron micrographs of influenza VLPs purified from the culture medium of infected cells. Bar = 50 nm. In the lower panel white arrows indicate the position of 5 nm gold particles labelling the HA spikes of the VLPs. B) Bluetongue virus VLPs. Left, stained SDS-PAGE of lysate from cells infected with AcMNPV expressing: Lane 1, BTV-1 VP5 (*p10 *locus); Lane 2, BTV-1VP2 (*odv-e56 *locus); Lane 3, BTV-1 VP3 (*polyhedrin *locus); Lane 4, BTV-1 VP7 (*gp37 *locus); Lane 5, Semipurified VLPs recovered from a virus coexpressing all 4 BTV proteins; M, marker lanes with proteins of defined mass. Right hand panels, electron micrographs of negatively stained BTV VLPs. Bar = 50 nm.

To confirm that the system could successfully express and correctly assemble protein complexes, two well defined virus-like particle (VLP) systems were used as models. VLPs were produced for influenza A virus by co-expressing the M1 and HA proteins of the SC35M strain of influenza [31]. M1 was first inserted at the *egt *locus and then, following removal of the bipartite cassette, the HA gene was inserted into the *p10 *locus (Fig. 4A). Expression of both proteins was confirmed by SDS-PAGE and Immunoblot analysis (Fig. 4A, left and middle panels) using antibody specific for Influenza A (H7N7). Furthermore, it was possible to purify influenza VLPs from the culture medium of *Sf*9 cells infected with the recombinant baculovirus. The surface of these VLPs had the characteristic spike appearance of an enveloped virus (Fig. 4A, right panel) and it was possible to label these spike structures using immunogold labelling with anti-influenza (H7N7) antibody.

The other VLP made was for the non-enveloped orbivirus, bluetongue virus. VLPs for this virus require the coassembly of four proteins (VP2, VP3, VP5 and VP7). Expression of recombinant protein was confirmed by SDS-PAGE analysis (Fig. 4B, left panel) and VLPs with the characteristic morphology of mature BTV particles could be purified from insect cells infected with recombinant baculovirus (Fig. 4B, right panel).

### Coexpression of eight genes to recover the mouse CCT complex

**Figure 5:**
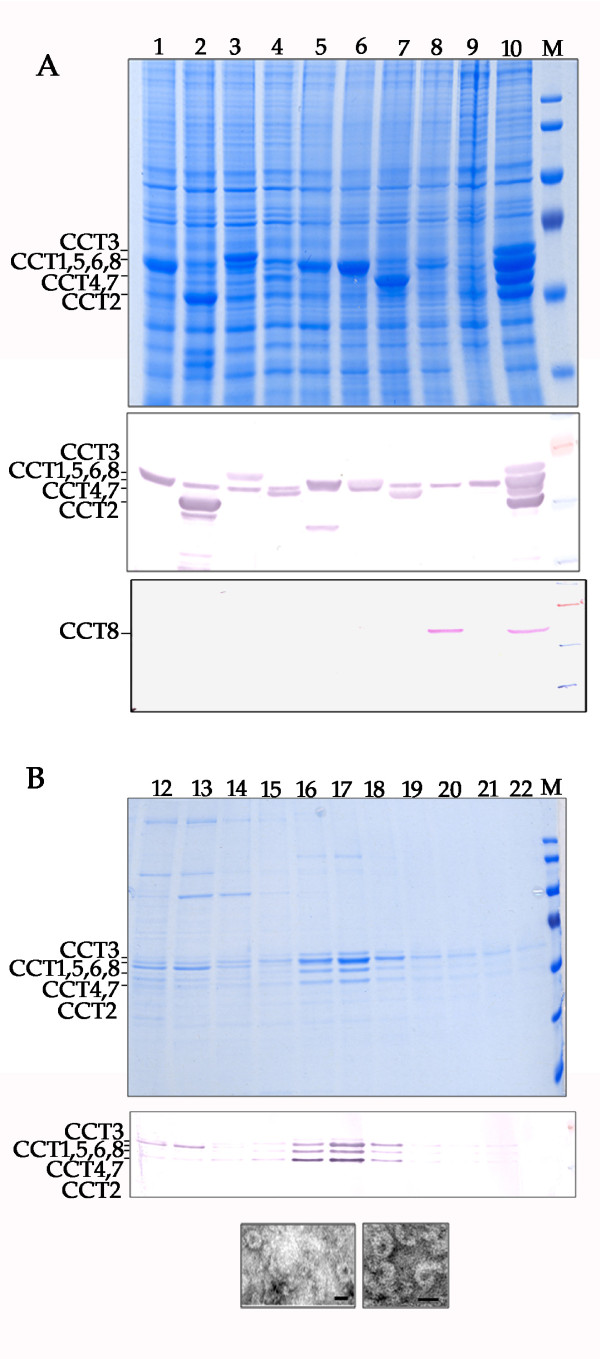
**Recovery of recombinant mouse CCT complex by co-expression of 8 proteins in the same insect cell**. A) Top panel, cell lysates from cells expressing mouse CCT subunits 1-8 (lanes 1-8, respectively). Lane 9, uninfected cells; Lane 10, lysate from cells coexpressing the CCT subunits from the same baculovirus genome; M, marker protein lane. Relative migration of CCT subunits is indicated on the left hand side of the gel. Loci used in the expression were CCT1-*polyhedrin*, CCT2-*p10*, CCT3-*egt*, CCT4-*39K*, CCT5-*gp37*, CCT6-*odv-e56*, CCT7-*ctx*, CCT8-*orf51*. Middle panel, Immunoblot using pan-specific antiserum against mouse CCT, sample order as in top panel. Bottom panel, Immunoblot using monospecific antiserum against mouse CCT theta (CCT8), sample order as in top panel. B) Fractions 12 to 22 (of 34) numbered from the top of a 10%-40%(w/w) sucrose gradient. CCT complex proteins peak in the expected ratio in fractions 16 and 17. Relative migrations expected for the CCT proteins are indicated on the left of the gel. Middle panel, immunoblot using pan-specific antiserum against mouse CCT subunits. Lower panel, electron micrographs of negatively stained sample from the 23.5S peak fraction collected from the sucrose gradient. Ring structures consistent with CCT complexes can be detected. Bar = 25 nm.

To extend our observations further, and to demonstrate the utility of the muli-locus expression system to the production of large mammalian protein complexes, the eight subunits of the mouse CCT chaperonin complex (CCT1 to CCT8, respectively) were each expressed from a different locus within the baculovirus genome (Fig. 5A.). All eight subunits were expressed and accumulated in insect cells to detectable levels, although there was a variation in the steady-state levels of protein between subunits. Expressed protein was detected with a polyclonal antibody against a conserved motif of the ATP-binding site of all CCT subunits (Fig. 5A middle panel). There was cross reaction between the antibody and some of the subunits of the endogenous insect CCT complex, and it was impossible to confirm the presence of CCT8. To confirm mouse CCT8 was expressed, a duplicate immunoblot was probed with a monospecific antibody to the CCT8 protein (Fig. 5A bottom panel). These data confirmed all eight CCT subunits were expressed using the multi-locus expression approach.

The native CCT chaperone complex consists of a hexadecamer arranged as two stacked rings of 8 subunits [32,33]. To confirm formation of a complex for the recombinant mouse CCT subunits, we performed rate-zonal centrifugation in 10-40% (w/w) sucrose gradients. The characteristic protein profile for the CCT complex was detected as a peak following centrifugation (Fig. 5B, top panel lanes 16-17). The identity of these proteins as CCT subunits was confirmed by immunoblotting (Fig. 5B, middle panel). The averaged migration of this complex in the gradient over 3 experiments was consistent with a sedimentation coefficient of 23.5 +/- 1.1S and was similar to that reported for the native mouse CCT complex[32]. Finally, fractions corresponding with the 23.5S recombinant mouse CCT complex were visualised by negative staining and electron microscopy (Fig. 5B, bottom panel). It was possible to visualise ring-like structures consistent with those reported with the mouse CCT[32].

## Discussion

This report demonstrates that lambda red recombination can be used to efficiently engineer high level expression of recombinant protein and protein complexes from the AcMNPV genome. Using a new bipartite selection system up to 88 fold improvement (p = 0.03) in recovery of recombinants was achieved. All putative recombinants recovered using the bipartite selection were subsequently confirmed to be correctly modified. This is a substantial improvement over the single antibiotic selection [21,28] which in our hands produced a high level of false positive recombinants (recombinants that were apparently chloramphenicol resistant but subsequently did not have the target insertion). Furthermore, there was no requirement to use PCR generated linear DNA for recombination, which avoids potential problems associated with mutations introduced by repeated PCR of coding regions. To our knowledge the bipartite selection in this report has not previously been applied to recombination in baculovirus, although there have been reports of the zeocin resistance gene alone being used for knock out of baculovirus genes by lambda red recombination[34].

By flanking the Zeo^R^-LacZα selection with self inactivating *loxP *sites it was possible to re-use the selectable markers and carry out iterative selection at multiple loci within the baculovirus genome (Fig. 2). This iterative process itself allows the potential for combining several different subunits into the same baculovirus genome in different combinations without having to generate a new multi-gene transfer vector for every combination of proteins that is to be expressed.

Despite the extensive protein expression work that has been undertaken in the baculovirus system most expression has focussed on replacement of the polyhedrin or *p10 *genes. Relatively little literature describes the use of alternative loci for the expression of recombinant protein [10,35-37]. This study identifies seven genetic loci (*ctx, egt, 39k, orf51, gp37, iap2 *and *odv-e56*), in addition to polyhedrin and *p10*, suitable for high level expression of foreign genes.

For the loci that did not give good expression, four (*orf11*, *v-fgf*, *pe *and *orf23*) also resulted in a reduced expression of a separate marker protein present in all recombinants. This suggests that these insertions resulted in impairment of functions essential for virus replication or gene expression. The low level expression of firefly luciferase expression in the *chiA *and *odv-e18 *insertion viruses was unexpected for different reasons. Other reports have recorded insertion of recombinant protein expression cassettes into the *chiA *locus [10,11]. It is possible that the reduced level of firefly luciferase expression from this locus in our experiments was due to effects on genes flanking the insertion. For *odv-e18*, recent reports based on deletion within the coding sequence of *odv-e18 *and the upstream flanking gene have suggested that this protein is essential for budded virus production and cell to cell movement [38,39]. In our experiments, where *odv-e18 *was inactivated by specific mutation of the start codon and by insertion of the firefly luciferase cassette, there was no evidence from renilla luciferase expression of impairment of the ability of this mutant virus to replicate or spread cell to cell. However, given the ~2 log reduction in firefly luciferase levels compared to virus without this insertion, we cannot rule out the possibility that a small population of virus in which the mutation had reverted was complementing the virus expressing the reporter gene.

To confirm the utility of the system for the expression of recombinant protein complexes two-protein and four-protein virus like particles (VLPs) were produced to influenza A and bluetongue virus, respectively. VLPs for both these viruses are effective immunogens against the respective pathogen [40-42] and thus represent potential biotechnological applications of the multi-locus expression approach. Both VLPs were formed normally and could be purified from the culture medium (for influenza VLPs), or cells (for BTV), of baculovirus infected cultures. To extend these observations further and to demonstrate the usefulness of the approach to low abundance mammalian protein complexes we expressed all eight subunits of the mouse CCT complex. This complex is usually purified from testes or by large scale fermentation culture of yeast [32,43,44]. However a reliable source of large quantities of correctly folded recombinant mammalian CCT complex was not previously available. It was possible to recover between 1 and 6 mg/L culture of 23.5S CCT complex. To our knowledge this is the first report of the successful expression of mammalian CCT chaperone from a heterologous system, and offers for the first time the possibility of mutational analysis of this essential machinery involved in cytoskeletal protein folding and cell cycle control.

In addition to providing the opportunity for assembly of multiprotein complexes, the multilocus system also offers the opportunity to test and manipulate relative expression of each subunit of a complex independently, without any need for recloning into different transfer vectors, and for swapping subunits between complexes, also without need for recloning. Thus, in terms of flexibility, it offers substantial improvement over the current approaches for baculovirus multi-protein complex expression.

## Conclusion

**1**. Use of bipartite selections can significantly improve selection of modified bacterial artificial chromosomes carrying baculovirus DNA. Furthermore this approach is sufficiently robust to allow routine modification of the virus genome. **2**. In addition to the commonly used *p10 *and polyhedrin loci, the *ctx, egt, 39k, orf51, gp37, iap2 *and *odv-e56 *loci in AcMNPV are all suitable for the high level expression of heterologous genes. **3**. Two protein, four protein and eight protein complexes including virus-like particles and cellular chaperone complexes can be produced using the new approach.

## Methods

### Cell Lines and Virus

*Sf9 *and *Sf*21 cells were cultured at 28°C in Insect-Xpress (Lonza, Basel, Switzerland) and TC100 media (Biochrom AG, Berlin, Germany), respectively. All cells were routinely cultured in the presence of penicillin (100 U)/streptomycin (100 μg/ml)/Amphotericin B (250 ng/ml) (Sigma-Aldrich Chemical Co., St. Louis, Mo.). TC100 medium was supplemented with 10% Foetal bovine serum. Viruses used in this study were based on the cloned AcMNPV genome [3]. For recombinants where protein was expressed from the polyhedrin locus, the BAC10:KO1629 bacmid [28] was used, for all other expressions a modified version of the bacmid bMON14272 [3] in which the expression of the *LacZα *fragment had been inactivated was used.

### Antibodies

Polyclonal anti influenza H7N7 antibody was a gift from H.D. Klenk (University of Marburg). The pan-CCT antibody, UM1, and the monospecific CCT8 antibody used were as described [45]. Anti rabbit immunogold antibody was purchased from Sigma-Aldrich. Negative staining was with 2% (w/v) uranyl acetate. All immunoblotting was performed using Immobilon P membrane (Millipore, Billerica, MA) according to the manufacturer's instructions.

### Construction of transfer vectors

Base expression and selection cassettes with the structure detailed in Fig. 1A, but without flanking AcMNPV were constructed in pBluescript SK (Stratagene, Cedar Creek, Texas). Transfer vectors targeting the different genetic loci within baculovirus were constructed by the general strategy of amplification and cloning of the corresponding region of the AcMNPV genome followed either by mutagenesis to introduce a restriction enzyme site, allowing insertion of the base expression and selection cassette, or digestion with restriction enzymes to introduce the same cassette. Details of the primers and restriction enzymes used for each locus are detailed in table 1. All constructs were designed such that a linear DNA fragment containing the AcMNPV flanking sequences and the expression and selection cassettes could be released by digestion with *BsaI*. Where possible, for each locus the expression and selection cassettes were inserted in both orientations and firefly luciferase monitored independently in each orientation. For protein expression experiments the coding sequence of each gene was amplified from a cDNA clone of the respective gene and cloned downstream of the polyhedrin promoter into the same vectors. For CCT the complete set of mouse CCT cDNAs [46] were used.

**Table 1:** Primers used for amplification of sequences for each AcMNPV locus used and restriction enzyme site used for insertion of reporter and expression cassettes.

Locus	Forward Primer	Reverse primer	Restriction enzymes used
*ctx*	CACTTGACTCGATTGCGCG	TATTTATTGTCTACATGAACACG	m*EcoRV
*orf11*	GTTGCACCTTTGACGAAGCGG	TCACAATCCATAACACACAACAGG	m* *EcoRV*
*egt*	ATGTGCGACCATTGTTGGGC	GTTGTCACATCTGACTACTCC	pd
*orf23*	AGAGTGCGTTAATCTGTACACC	ATCATAGGGTACAACACAGG	*StuI/SfoI*
*v-fgf*	CCGGCAAAATCAAAGCGAGC	GATTACACGTGACATTTACGATGG	m* *EcoRV*
*39k*	CCTGGTAATTTTTGACCACG	CGCAGCAATTCCAGCGAGC	m* *EcoRV*
*orf51*	AAATGACTAGACAAGAAATTGCC	AGTTGTACAAATCACAAATATAAAAG	*BmgBI*
*gp37*	ATTGACGGGCCGTCGGCACG	CGATCATGCAAAAGTACATGC	m* EcoRV
*iap2*	CCGCGGCTAAGCGTTAAACC	TTCGAATACGTGTGTCGTTTAATTTGC	*BstBI/SacII*
*chiA*	TAAACGCTCCGACTCTGTGG	CGAGGGCCGCGGCCAGTGGGTC	pd
*pe*	GCATTTTTCCAATGTGGTAGACG	CTTTAGCGGTTTCCAACGCC	m* EcoRV
*odv-e18*	TCTCAAACACGGTGCCTGC	TCGTTGGTTTCAGTGACCAC	m* *EcoRV*
*odv-e56*	CAACATGACGCCGCTGCCG	TTATCGAGGGGCCGTTGTTG	m* *EcoRV*

m*EcoRV, indicates that an *EcoRV *site was engineered by site directed mutagenesis using the quickchange site directed muagenesis system (Stratagene) according to the manufacturer's directions. Pd indicates that a deletion was engineered by PCR. In the case of the egt locus this deletion removes the equivalent of nt 11622-12474 nt in the AcMNPV genome. For *chiA *the engineered deletion removes the *chiA *and cathepsin genes and is positioned nt 105461-107946 nt in the AcMNPV genome.

### Luciferase assays

All luciferase assays were completed using the Dual Luciferase Reporter Assay system (Promega, Madison, USA) using a Turner Biosciences 20/20 n luminometer according to the manufacturer's instructions.

### Lambda red recombination in *E. coli*

All bacmids were maintained in the *E. coli *strain EL350 [47] which was grown at 32°C. Expression of the λ *bet, exo *and *gam *genes in this lysogen were induced by incubation at 42°C for a 30 minute period immediately harvesting cells for the preparation of batches of cells for electroporation. Once prepared, electrocompetent cells were stored at -80°C prior to use. For lambda red recombination 30 ng linear DNA was introduced into recombination competent cells by electroporation. Cells were grown for two hours at 32°C and then plated under Kanamycin (50 μg/ml) Zeocin (15 μg/ml) and IPTG/X-gal (0.2 mM and 40 μg/ml respectively). For modification of BAC10:KO1629 chloramphenicol selection (15 μg/ml) was also included. Plates were incubated at 32°C and blue colonies picked to seed liquid cultures after 24 hours. All preparation of DNA was performed according to standard molecular techniques [48].

### Cre recombination in *E. coli*

Expression of Cre recombinase in EL350 was induced by treatment of mid-log phase cultures with 0.1% Arabinose for 2 hours followed by a change of media and outgrowth for 16 hours at 32°C. Cells were then plated on selective media containing IPTG (0.2 mM), X-Gal (40 μg/m) and kanamycin (50 μg/ml). White colonies were re-streaked onto the same selection after 24 hours and then used to seed liquid cultures.

### Purification of VLPs

Bluetongue VLPs were purified from infected *Sf9 *cells as described [7]. Influenza A (HA/M1) VLPs were purified from the culture medium of infected *Sf*9 cells at 72 hours post infection. Culture medium was clarified by spinning at 9500 × *g *for 10 minutes at 4°C before being spun onto a 60% w/v sucrose cushion for 1 hour at 90,000 × *g *at 4°C in a SW28 rotor to concentrate VLPs. Material on top of the sucrose cushion was then diluted six fold in PBS and loaded onto a 20%-30%-60% (w/w) sucrose step gradient before being spun again for 1 hour at 90,000 × *g *at 4°C in a SW28 rotor. Material at the 30-60% sucrose interface contained the particles shown in Fig. 5.

### Purification of CCT

*Sf*21 cells were infected with recombinant baculovirus at multiplicity of infection of 3 and harvested at 48-72 hours post infection. Cells were washed in PBS and lysed at room temperature in lysis buffer (50 mM HEPES pH8.0, 100 mM NaCl, 0.05% (v/v) Tergitol NP-9). Nuclei were pelleted at 1000 × *g *for 5 minutes then cell debris pelleted at 9500 × *g *for 10 minutes at 4°C. Clarified cell lysate was spun at 100,000 × *g *at 4°C for 1 hour then serially filtered through 0.5 μm, 0.2 μm and 0.05 μm filters (Pall, East Hills, NY and Spectrum Laboratories, Breda, The Netherlands). Filtered lysate was concentrated using a centriplus filtration device (Millipore, Billerica, MA) with a 100 kDa cut-off. Concentrated lysate was loaded onto 10-40% (w/w) continuous sucrose gradients in 50 mM Hepes pH8.0, 100 mM NaCl and spun for 16 hours at 4°C at 30,000 rpm in a SW40 rotor. Following centrifugation 500 μl fractions were collected from the bottom of the tube.

## Authors' contributions

**RN **conceived the project, designed the experiments, carried out the research, and wrote the paper. **MS, MB **and **CC **made transfer plasmids for the expression of BTV VP3, VP5 and VP7, respectively. **KW **provided cDNA and antibodies for mouse CCT. He also provided substantial guidance on the best purification strategies for this protein complex. **PR **contributed to the design of experiments, provided expertise for purification of bluetongue VLPs and secured funding for the project. All authors were involved in the revision of the draft manuscript and have agreed the final content.

## Supplementary Material

Additional file 1 **Figure S1**. PDF file containing supplementary figure. Click here for file
