# Supplementary material for: Multigene expression of protein complexes by iterative modification of genomic Bacmid DNA
Source: BMC Mol Biol. 2009 Sep 2;10:87. doi: 10.1186/1471-2199-10-87 (PMC2749033; doi:10.1186/1471-2199-10-87)
Supplement: Additional file 1 — Figure S1. PDF file containing supplementary figure. [file 1471-2199-10-87-S1.pdf]

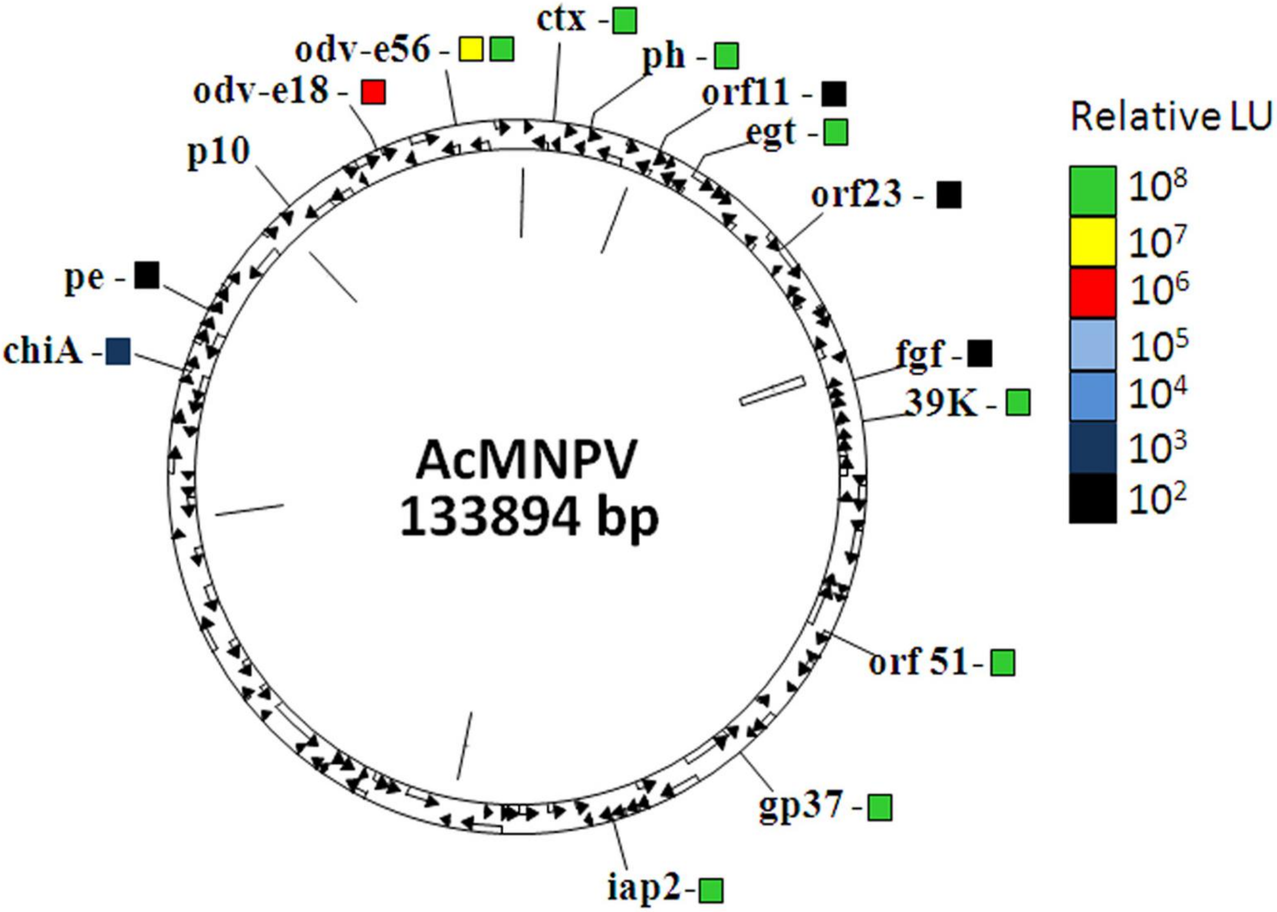

Supplementary Fig. 1. Relative expression of foreign genes inserted at different sites in the AcMNPV genome. Cartoon showing the position of loci tested for expression of polyhedrin promoter firefly luciferase reporter cassettes in the AcMNPV genome. The relative luciferase activity of lysate from infected cells with virus expressing the firefly luciferase gene at each locus is indicated.

Noad *et al*., 2009
